# *In Vitro* Assessment of Cytotoxicity, Antioxidant, and Anti-Inflammatory Activities of *Ricinus communis* (Euphorbiaceae) Leaf Extracts

**DOI:** 10.1155/2014/625961

**Published:** 2014-11-16

**Authors:** Vhutshilo Nemudzivhadi, Peter Masoko

**Affiliations:** Department of Biochemistry, Microbiology and Biotechnology, University of Limpopo, Turfloop Campus, Private Bag X1106, Sovenga 0727, South Africa

## Abstract

*Ricinus communis* has been utilized traditionally as medicine to treat inflammatory related diseases including wounds, sores, and boils. The leaves of *R. communis* were sequentially extracted with *n*-hexane, dichloromethane, acetone, and methanol using serial exhaustive extraction method. Antioxidant activity of all crude extracts was quantitatively measured against 2,2′-azino-bis (3-ethylbenzothiazoline-6-sulphonic acid) free radical molecules using ABTS^+^ assay. Cytotoxic effect and anti-inflammatory activity of *R. communis* leaves extracts were evaluated on Human Caucasian skin fibroblast and Raw 264.7 macrophage cell lines, respectively. Methanol extract had the highest percentage free radical (ABTS^+^) scavenging activity of 95% at 2.50 mg/mL, acetone 91%, dichloromethane 62%, and hexane the least (50%). Percentage scavenging activity of ABTS^+^ free radical molecules increases with increase in concentrations of the plant extracts. Hexane and dichloromethane extracts had more than 90% cell viability at 100 *µ*g/mL after 24 and 48 hours of exposure. Methanol extract had LC_50_ of 784 *µ*g/mL after 24-hour exposure, hexane had 629.3 *µ*g/mL and dichloromethane 573.6 *µ*g/mL, and 544.6 *µ*g/mL was the lowest with acetone extract. The study present the first report on the scavenging activity of *R. communis* leaf extracts against ABTS^+^ radicals and cytotoxic effects on human Caucasian skin fibroblast cell lines.

## 1. Introduction

Since ancient times, medicinal plants have played a vital role in preserving human health. The use of medicinal plants has increased in rural areas and developing countries. This is due to lack of hospitals, poverty, and an increased demand of inexpensive medicines [1]. Medicinal plants continue to provide humanity with new remedies. It is therefore important to explore medicinal plants for their safety, quality, toxicity, appropriate amount of plant materials to use, and efficacy. Natural products of plants possess several biological activities including antioxidant and anti-inflammatory activity [2, 3]. Medicinal plants are rich in active phytochemical compounds with various biological activities. Researchers are highly interested in studying plants with the aim of isolating novel active drugs to replace synthetic drugs present in the market. The availability of these plants constituents provides a source of natural drugs for modern medicine [4]. Hence, cytotoxic level of medicinal plants must also be evaluated against host cells. The safety of plants as a potential therapeutically agents must be ascertained and the side effects should be acceptable to the host. Bioactive compounds with no or less toxic effect to the host are the good candidates for formulation of drugs [5].

Antioxidants play an important role in neutralizing free radical species which are produced as end or by-products of normal biochemical reactions in normal system [6]. High amounts of free radical molecules cause oxidative stress in cells which result in damaging essential macromolecules including DNA, lipids, and proteins. The damage of macromolecules leads to inflammation and many degenerative conditions such as Parkinson's diseases, atherosclerosis, aging, immunosuppression, ischemic heart disease, diabetes, hair loss, membrane lipid peroxidation, and decreased membrane fluidity [3, 6–8]. Reactive oxygen species are also reported as carcinogenic and mutagenic agents [9]. Signs of inflammation are swollen joints, joint pain, stiffness, and loss of joint functions. Nonsteroidal anti-inflammatory drug (NSAIDs) such as ibuprofen and naproxen are anti-inflammatory drugs currently used for treatment of inflammation. These drugs are known to cause severe side effects in the body such as heart attacks and stroke [10].

Plant constituents are responsible for both free radicals scavenging and anti-inflammatory activity. Secondary metabolites are responsible for biological activities of plants including terpenoids [11], phenolic compounds (flavonoids, phenolic acids, quinones, coumarins, lignans, stilbenes, tannins), and nitrogen compounds (alkaloids, amines, and betalains) and carotenoids [12]. Iqbal et al.'s [13] reports are available which suggest that phenolic compounds are the potent antioxidant compounds from medicinal plants.

*Ricinus communis* (Castor oil plant) belongs to the family Euphorbiaceae. Castor oil plant is originally from Africa and is now found in all tropical countries [14]. Leaves, barks, seeds, roots, and oil of the plant have been traditionally used for many purposes throughout the world. The leaf, root, and seed oil of the plant have also been used for treatment of inflammation and liver disorders in India [15]. In Tunisia,* R. communis* is used as a contraceptive herbal drug, for treatment of cold and tumors, as a hypoglycemic, and as a laxative.* R. communis *is well known for its biological activities, most important of which are hepatoprotective, laxative, antidiabetic, and antifertility activities [16]. Literature search revealed that essential oils from* R. communis* have been reported to have potential antimicrobial, anticarcinogenic, antioxidant, and antidiabetic activities [17–19]. The aim of this study was to evaluate the cytotoxic effects of* R. communis* extracts against Human Caucasian skin fibroblast (BUD-8) cell line as well as its antioxidant activityusing 2,2′-azino-bis (3-ethylbenzothiazoline-6-sulphonic acid) (ABTS) free radicals and anti-inflammation activity on the Raw 264.7 macrophages cell line.

## 2. Methods and Materials

### 2.1. Plant Collection and Processing

Leaves were collected in summer of 2011 from University of Limpopo (Turfloop campus), South Africa. Voucher specimens in the garden Herbarium and tree labels verified the identity of the plant. Plant was confirmed by Dr. Brownyn Egan (Herbarium) and the accession detail was UNIN 11352. The plant was selected based on the high antioxidant activity demonstrated in previous* in vitro *study [20]. Leaves were separated from twigs and dried at room temperature. The dried plant materials were milled to fine powder and stored at room temperature in closed bottles in the dark until use for the extraction.

### 2.2. Extraction Procedure

Dried plant materials were sequentially extracted by mixing 10 g of plant material with 100 mL of hexane, dichloromethane, acetone, and methanol in glass bottle. Bottles were vigorously shaken at a speed of 300 rpm, overnight. Plant residues were allowed to settle and the supernatant was filtered and evaporated using a rotary evaporator (BÜCHi Labotec rotavapor model R-205, Germany) and transferred into preweighed labeled glass beakers. The process was repeated three times to exhaustively extract the plant material and the extracts for each solvent were combined. The solvent was removed under a stream of cold air at room temperature. Plant extracts were reconstituted using acetone to a final concentration of 10 mg/mL.

### 2.3. Analysis of Extracts by TLC

Ten microliters of 10 mg/mL plant extracts were loaded on a thin layer chromatography plates. Three separation systems of varying polarities were used to analyse plant extracts by thin layer chromatography (TLC) (Fluka, silica gel F_254_ plates): benzene : ethanol : ammonium hydroxide (BEA) (36 : 4 : 0.4), chloroform : ethyl acetate : formic acid (CEF) (20 : 16 : 4), and ethyl acetate : methanol : water (EMW) (20 : 10.8 : 8). Chromatograms were examined under ultraviolet light (254 and 365 nm) and sprayed with freshly prepared vanillin spray reagent (0.1 g vanillin, 28 mL methanol, and 1 mL sulphuric acid) to visualise separated compounds. The plates were carefully heated at 110°C for optimal colour development [21].

### 2.4. Antioxidant Activities

#### 2.4.1. Qualitative 2,2-Diphenyl-1-picrylhydrazyl Assay on TLC

Thin layer chromatography plates were used to separate crude extracts as described in Section 2.3. To detect antioxidant activity, chromatograms were sprayed with 0.2% (w/v) 2,2-diphenyl-1-picrylhydrazyl (DPPH) (Sigma) in methanol as an indicator. Yellow zones appearing on the chromatograms indicate the presence of scavenging activity of free radicals by compounds presence in plant extracts [22].

#### 2.4.2. Quantitative Antioxidant Assay Using ABTS^+^

A stock solution of 7 mM of ABTS^+^ was prepared according to van den Berg et al. [23] by mixing 8 mg of ABTS with 1 mL of water. ABTS free radicals were produced by reacting equal volume of ABTS stock solution with 13.2 mg in 10 mL of (2.45 mM) potassium sulphate. The solution was prepared and incubated for 12–16 hours in the dark at room temperature until the reaction was complete and stored at 4°C until use. Serial dilution method was used to prepare different concentrations of crude extracts (hexane, dichloromethane, acetone, and methanol). ABTS^+^ was diluted with water for equilibration of absorbance 0.70 (±0.02) at 734 nm and appropriate blank was used without test samples. Hundred microliters of ABTS^+^ was added to 100 *μ*L of 2.50, 1.25, 0.63, 0.16, 0.08, and 0.04 mg/mL concentrations of crude extracts. The absorbance reading was taken after 6 minutes following the reaction. All determinations were carried out in triplicate and calculated as follows:

(1) %  Total  antioxidant  activity   =Absorbancecontrol−AbsorbancesampleAbsorbancecontrol×100.

### 2.5. *In Vitro* Cytotoxicity Using MTT Assay

Cell viability was assessed according to Mosaddegh et al. [24] on the Human Caucasian skin fibroblast (Bud-8) cell line. The proliferation rates of Bud-8 cell line after treatment with plant extracts were determined by the 3-(4,5-dimethylthiazol-2-yl)-2,5-diphenyl tetrazolium bromide (MTT) assay. MTT is reduced by mitochondrial dehydrogenases to the water-insoluble pink compound formazan, depending on the viability of cells. Cells were maintained in the Dulbecco's Modified Essential Medium (DMEM) supplemented with 10% fetal bovine serum (FBS) and 1x Penicillin-Streptomycin-Neomycin (PSN). Hundred microliters of cells (4 × 10^4^ cells/mL) was seeded in 96-well plates and incubated at 37°C, 5% carbon dioxide for 24 hours. After 24 hours of incubation, the cells were treated with 100 *μ*L of 100, 200, 300, 400, and 500 *μ*g/mL of plant extracts (hexane, dichloromethane, acetone, and methanol). Plates were incubated at 37°C, 5% CO_2_ for 24 and 48 hours. After incubation, morphology of cells was examined under microscope. Twenty microliters of MTT solution (5 mg/mL) (Sigma) was added to each well. The plates were further incubated for 2 to 4 hours and the medium was removed. Formazan crystals were dissolved with 100 *μ*L of dimethyl sulfoxide (DMSO). The absorbance was measured at 560 nm and percentages of cell viability and LC_50_ of cells were calculated:

(2) $$\begin{array}{c} \%\;\text{cell}\;\text{viability}\;=\frac{{\text{Absorbance}}_{\text{sample}}}{{\text{Absorbance}}_{\text{control}}}\times 100, \end{array}$$

where Absorbance_control_ is the absorbance of cells treated with DMSO 1% and Absorbance_sample_ is the absorbance of cells treated with test sample.

### 2.6. Anti-Inflammatory Activity Using PhagoBurst Assay

Two hundred microliters of cells (Raw 264.7 macrophages) was transferred to a coverslip in a multiwell plate. The cells were incubated at 37°C, 5% CO_2_ overnight, to allow cells to attach. Cells were treated with 100 *μ*L of plant extracts (100 *μ*g/mL of hexane, dichloromethane, acetone, and methanol) and 10 mg/mL of LPS for 24 hours. Two microliters of PMA was also added for 30 minutes. After incubation, the medium was aspirated. Cells were stained with 50 *μ*L of 10^−5 ^M of H_2_DCF-DA and incubated for 20 minutes in the dark. Cells were stained for the second time with 50 *μ*L of 20 *μ*g/mL, 4′,6-diamidine-2-phenylindole (DAPI) and further incubated for 20 minutes in the dark. Staining solutions were removed and 3.7% paraformaldehyde was added to a coverslip to fix the cells. Coverslips were mounted to a microscope slide and examined under fluorescence microscope.

## 3. Results and Discussion

**Figure 1:**
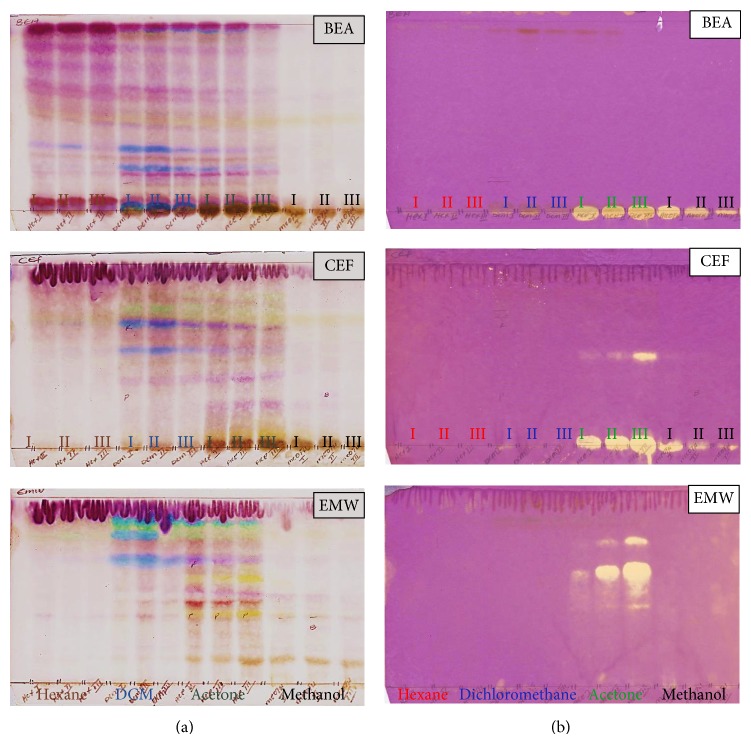
Chromatograms of* R. communis* leaves extracted with hexane, dichloromethane (DCM), acetone, and methanol, in lane from left to right, separated with three solvent systems, BEA, CEF, and EMW from top to bottom and sprayed with vanillin-sulphuric acid reagent (a) and 0.2% DPPH in methanol as an indicator (b); yellow spots against purple background indicate antioxidant activity.

**Figure 2:**
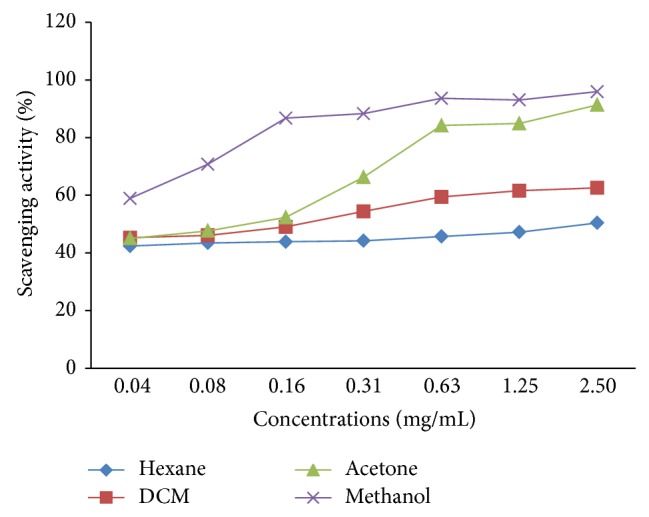
Percentage scavenging activity of ABTS^+^ free radicals with respect to increasing concentration of* R. communis* crude extracts; hexane, dichloromethane (DCM), acetone, and methanol extracts.

Medicinal plants have unlimited capacity to synthesis of bioactive compounds that are effective and have fewer side effects compared to synthetic drugs. Bioactive compounds from plants have shown over the years to have various biological activities. Scientists have developed a greater interest of using these compounds in formulation of new and novel drugs, because of their biological activities and reliability [25]. Crude extracts were separated with three mobile phases and sprayed with vanillin-sulphuric acid to visualize phytochemical compounds present (Figure 1(a)). To qualitatively analyse crude extracts for antioxidant activity, chromatograms were sprayed with 0.2% DPPH with yellowish spots indicating the presence of antioxidant compounds (Figure 1(b)). Acetone extracts revealed strong antioxidant compounds against 0.2% DPPH free radicals in EMW solvent system while hexane, DCM, and methanol extracts did not. Acetone and methanol extracts showed antioxidant compounds in CEF and EMW solvent systems even though active compounds were not separated due to their polarity relative to the solvent systems. Iqbal et al. [13] also reported on the potent antioxidant activity of the aerial part of* R. communis.* So far there is nothing reported on the scavenging activity of* R. communis* leaf extracts against ABTS free radicals. Antioxidant activity of hexane, dichloromethane, acetone, and methanol extracts of* R. communis* was also quantified using ABTS^+^ decolonization method. Addition of antioxidants to free radicals reduced the blue chromophores ABTS^+^ to colorless. Methanol extract had the highest percentage free radical (ABTS^+^) scavenging activity of 95% at 2.50 mg/mL, acetone 91%, dichloromethane 62%, and hexane 50%, the least (Figure 2). Polar solvents seem to extract compounds responsible for antioxidant activity of* R. communis*. ABTS^+^ decolorization method is used for the screening of antioxidant activity of plant extracts and is applicable to both lipophilic and hydrophilic antioxidants [26]. Percentage scavenging activity of both ABTS and DPPH free radical molecules increases with the increase of concentrations of the extracts. The present study suggests the leaves of* R. communis* as good source of antioxidant compounds to scavenge both ABTS and DPPH free radicals. Scavenging activity of ABTS free radical molecules has never been reported before on the leaves of* R. communis*, although it has previously been reported in the seeds of the* R. communis* by Surveswaran et al. [27].

There are other methods which are used to test for antioxidants, like ferric reducing antioxidant power (FRAP) method, electron spin resonance spectrometry and Phycoerythrin assay. FRAP method is based on the reduction of a ferroin analog, the Fe^3+^ complex of tripyridyltriazine Fe(TPTZ)^3+^, to the intensely blue coloured Fe^2+^ complex Fe(TPTZ)^2+^ by antioxidants in acidic medium. Results are obtained as absorbance increases at 593 nm and can be expressed as micromolar Fe^2+^ equivalents or relative to an antioxidant standard. This method is automated; however, the measured reducing capacity does not necessarily reflect antioxidant activity. It provides instead a very useful “total” antioxidant concentration, without measurement and summation of the concentration of all antioxidants involved.

Electron spin resonance (ESR) spectrometry is the only analytical technique that can specifically detect the free radicals involved in autoxidation and related processes. The assay is intrinsically sensitive to stable free radicals such as di-tert-butyl nitroxide. ESR is unfortunately insensitive to detecting the reactive, short-lived free radicals involved in autoxidation (lifetimes vary from 10-9 s for the hydroxyl radical to several seconds for the peroxyl radical). Phycoerythrin assay is particularly useful in screening for compounds that protect against damage by chelating metal ions necessary for site-specific formation of the radical species. The inhibition of oxidation by an antioxidant can be examined by the retardation of the loss of fluorescence, with the inhibition being proportional to the antioxidant activity. Results can be calculated using the differences in areas under the phycoerythrin decay curves between the blank and a sample and are expressed in Trolox equivalents.

**Figure 3:**
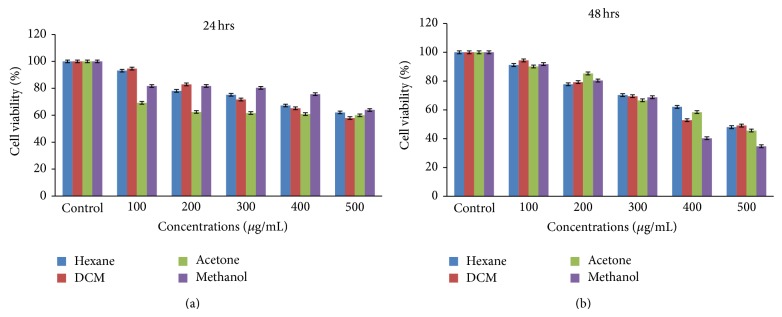
(a) Cytotoxicity effect of* R. communis* leaf extracts against Bud-8 cell line after 24 hours of exposure using MTT assay at the concentration ranging from 100 to 500 *μ*g/mL. (b) Cytotoxicity effect of* R. communis* leaf extracts against Bud-8 cell line after 48 hours of exposure using MTT assay at the concentration ranging from 100 to 500 *μ*g/mL.

A major concern about bioactive compounds from plants is that some of these compounds are toxic to our normal system; therefore safety is critical in development of novel drugs [5]. In this study cytotoxic effect of hexane, dichloromethane, acetone, and methanol extracts of* R. communis* at concentration ranging from 100–500 *μ*g/mL was investigated on Human Caucasian skin fibroblast (Bud-8) cell line by using MTT assay. Effect of* R. communis *extracts on Bud-8 cell line was evaluated after 24 (Figure 3(a)) and 48 hours (Figure 3(b)) of cell exposure to the extracts. Percentage cell viability was calculated by measuring the absorbance of pink colour formazan formed from reduction of MTT solution by the presence of mitochondrial dehydrogenase in viable cells. All crude extracts were toxic to Bud-8 cell line at the higher concentration of 500 *μ*g/mL. Morphology of cells was also altered from its normal shape of fibroblast to oval shape, because of the toxic effect of* R. communis* extracts to the cells. However, at the lower concentrations (100 *μ*g/mL), hexane and dichloromethane extracts had more than 90% cell viability after 24 and 48 hours of exposure. Acetone and methanol extracts had less than 90% cell viability. Cell viability decreased with the increase in concentrations of plant extracts. Higher concentrations of* R. communis* extracts were observed to be toxic on Bud-8 cell line. Rana et al. [28] reported the toxic effect of ricin isolated from the seeds of* R. communis*. Ricin kills the cells by disrupting protein synthesis in the cells. Ricin was the only compound isolated from* R. communis* seeds that was reported to have high toxic effect on mammalian cells. Leaves of* R. communis *also contain ricin but in lower concentration. The present finding presents the first report on the cytotoxicity effects of* R. communis* leaf extracts against Human Caucasian skin fibroblast cell lines.

The LC_50_ of* R. communis* extracts on Bud-8 cell line was also calculated for each extract after 24 and 48 hours of exposure (Table 1). LC_50_ indicates the lowest concentration of plant extracts that inhibits 50% of cells. Methanol extract had high LC_50_ of 784 *μ*g/mL after 24-hour exposure, followed by hexane (629.3 *μ*g/mL) and dichloromethane (573.6 *μ*g/mL), and the lowest was in acetone extract (544.6 *μ*g/mL). Morobe et al. [5] also reported low toxic effect of methanol extracts on MAGI CCR5+ cell lines after 24 hours. The LC_50_ for all crude extracts decreased after 48 hours of exposure. Hexane had the highest LC_50_ of 495 *μ*g/mL and methanol had the lowest LC_50_ of 387.1 *μ*g/mL. In general, low LC_50_ values represent high toxicity. Extracts with high LC_50_ are preferable to work with, because of their lower toxicity effects on the host cells. The cytotoxicity of the oil of* R. communis* was also reported with LC_50_ values less than 2.63 mg/mL for HeLa cell line [19]. The CC_50_ values of* R. communis* leaf extracts on Vero cell line was reported at 16.5 mg/mL [29].

In Cell Proliferation Reagent WST-1 assay the Cell Proliferation Reagent WST-1 is a ready-to-use colorimetric assay for the nonradioactive quantification of cellular proliferation, viability, and cytotoxicity. Sample material is either adherent or suspension cells cultured in 96-well microplates. The assay is based in the enzymatic cleavage of the tetrazolium salt WST-1 to formazan by cellular mitochondrial dehydrogenases present in viable cells. In principle, WST-1 works similarly to MTT by reacting with the mitochondrial succinate-tetrazolium reductase forming the formazan dye. The WST-1 reagent produces a water-soluble formazan rather than the water-insoluble product of the MTT assay. It can be used for measurement of cell proliferation in response to growth factors, cytokines, mitogens, and nutrients and analysis of cytotoxic and cytostatic compounds, such as anticancer drugs and other pharmaceutical compounds.

**Figure 4:**
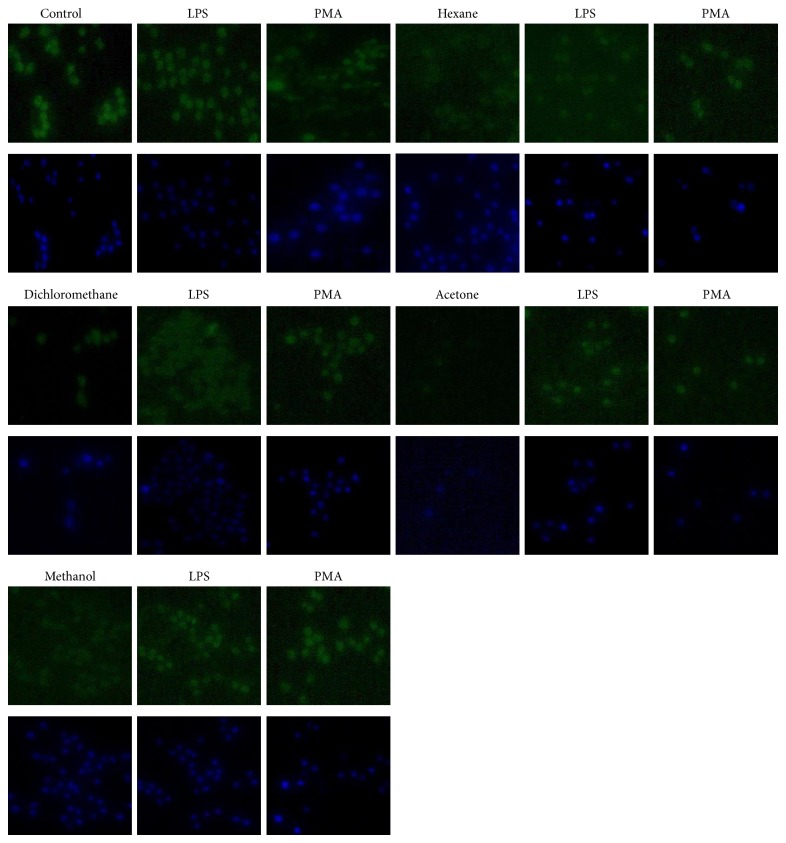
Effect of* R. communis* extracts on Raw 264.7 macrophages cells in response to oxidative stress and inflammation. Reactive oxygen species were induced by LPS and PMA stimulants. Cells were fixed and stained with H_2_DCF-DA to measure the amount of free radical molecules produced in the cells (green) and DAPI to locate the nucleus in the cells (blue).

Roots extracts of* R. communis* have been reported to neutralize free radical molecules and anti-inflammatory activity. The same study suggested that the anti-inflammatory activity of roots extracts of* R. communis* was because of the presence of flavonoids [30]. The fluorogenic cell permeant, H_2_DCF-DA (green cells), was used to measure the level of reactive oxygen species (ROS) in activated Raw 264.7 macrophage cells induced by LPS and PMA stimulants (Figure 4). High level of reactive oxygen species tends to attack macromolecules and this facilitates cells to undergo oxidative stress and inflammatory response. Antioxidant compounds scavenge the free radical molecules by donating one electron or proton to a molecule. High intensity of green colour is the indication of high level of free radical molecules which can lead cells to undergo oxidative stress and inflammation. Reactive oxygen species act as a mediator to regulate cytokines production through activation of the transcription factors, such as NF-*κ*B. This suggests the direct link between ROS and other cytokines to initiate inflammatory response [31]. When dihydrodichlorofluorescein diacetate (H_2_DCF-DA) penetrates through the plasma membrane of cells and is then deesterified to a hydrophilic alcohol dihydrodichlorofluorescein (H_2_DCF), it is oxidized to fluorochrome DCF (2,7-dichlorofluorescein) by the presence of reactive oxygen species that fluoresce green when excited with blue light. The brightness of DCF fluorescence reflects the level at which reactive oxygen species are present in the cell [32]. A reduced internal H_2_DCF fluorescence intensity is an indication of reduced level of free radical molecules by antioxidant compounds extracted from* R. communis* leaves. Combination of hexane and acetone extracts with PMA and LPS stimulants depicts low fluorescence intensity as compared to PMA and LPS stimulated cells. Hexane, acetone, and methanol extracts were observed to reduce the level of ROS formation in cells while dichloromethane extract did not. Methanol extracts of* R. communis* have been reported to contain flavonoid, a constituent that has been reported as the anti-inflammatory agent of medicinal plants [33]. The root extracts of* R. communis* have been reported for their strong anti-inflammatory compounds [30]. The possibility that active constituent present in the roots may be responsible for observed activity in the leaves may not be ruled out. Roots and leaves materials may contain similar constituents at varying concentrations.

The Griess assay was not considered because of its limitations. This assay can only be used to measure the nitric oxide excluding other free radical molecules. In Griess assay nitric oxide (NO) is a molecular mediator of many physiological processes including inflammation. Griess assay is a colorimetric assay to measure the levels of nitrite oxide in aqueous solution. Griess assay involves the use of the Griess diazotization reaction to spectrometrically detect nitrite formed by the spontaneous oxidation of NO under physiological conditions. The Griess reaction can also be used to analyze nitrate via its catalytic reduction to nitrite.

## 4. Conclusion

The leaves of* R. communis* revealed strong antioxidant activity in both assay tests. Although some studies have reported on the cytotoxicity effects and anti-inflammatory activity of* R. communis* leaf extracts on other cell lines, this is the first study to report on the cytotoxicity effect and anti-inflammatory activity of* R. communis* leaves on Human Caucasian skin fibroblast and Raw 264.7 macrophage cell lines, respectively. Crude extracts of* R. communis* leaves revealed low toxic effect on Bud-8 cell line at lower concentrations while at high concentrations the extracts are toxic. The present study also agrees with previous findings indicating the potent anti-inflammatory activity of* R. communis* using other plants parts. The study serves as a scientific proof for the use of* R. communis* leaves in traditional medicine for treatment of inflammatory related diseases. Further studies are required to isolate anti-inflammatory compounds. Traditional healers will be advised to use the leaves instead of uprooting the plants.

## Figures and Tables

**Table 1:** The LC_50_ of *R. communis* extracts on BUD-8 cell lines after 24 and 48 hours of exposure.

Extracts	LC_50_ (*µ*g/mL)	
	24 hours	48 hours
Hexane	629.3	495
Dichloromethane	573.6	467
Acetone	544.5	471
Methanol	784	387.1
